# *MUTYH *Gln324His gene polymorphism and genetic susceptibility for lung cancer in a Japanese population

**DOI:** 10.1186/1756-9966-28-10

**Published:** 2009-01-22

**Authors:** Aiko Miyaishi, Kayo Osawa, Yasunori Osawa, Natsuko Inoue, Kana Yoshida, Mayumi Kasahara, Akimitsu Tsutou, Yoshiki Tabuchi, Kazuo Sakamoto, Noriaki Tsubota, Juro Takahashi

**Affiliations:** 1 Faculty of Health Sciences, Kobe University Graduate School of Medicine, Kobe, Japan; 2 Clinical Laboratory, Otemae Hospital, Osaka, Japan; 3 Osaka Cancer Immuno-Chemotherapy Center, Osaka, Japan; 4 Department of Surgery, Yoshida Ardent Hospital, Kobe, Japan; 5 Department of Radiology, Joyo Ejiri Hospital, Himeji, Japan; 6 Department of Thoracic Surgery, Hyogo Medical Center for Adults, Akashi, Japan; 7 Department of Thoracic Oncology, Hyogo College of Medicine, Nishinomiya, Japan

## Abstract

**Background:**

Genetic polymorphisms of DNA repair enzymes in the base excision repair (BER) pathway, may lead to genetic instability and lung cancer carcinogenesis. We investigated the interactions among the gene polymorphisms in DNA repair genes and lung cancer.

**Methods:**

We analyzed associations among *OGG1 *Ser326Cys and *MUTYH *Gln324His gene polymorphisms in relation to lung cancer risk using PCR-RFLP. The study involved 108 lung cancer patients and 121 non-cancer controls divided into non-smokers, smokers according to pack-years smoked in Japanese.

**Results:**

The results showed that the *MUTYH His/His *genotype compared with *Gln/Gln *genotype showed an increased risk for lung cancer (adjusted odds ratio [OR] 3.03, confidence interval [95%CI], 1.31–7.00, p = 0.010), whereas there was no significant increase for the *Gln/His *genotype (adjusted OR 1.35, 95%CI 0.70–2.61, p = 0.376). The *MUTYH His/His *genotype was at a borderline increased risk for both adenocarcinoma and squamous cell carcinoma (adjusted OR 2.50, 95%CI 0.95–6.62, p = 0.065 for adenocarcinoma; adjusted OR 3.20, 95%CI 0.89–11.49, p = 0.075 for squamous cell carcinoma, respectively). However, the *OGG1 Ser/Cys *or *Cys/Cys *genotypes compared with the *Ser/Ser *genotype did not have significantly increased risk for lung cancer, containing either adenocarcinoma or squamous cell carcinoma. The joint effect of tobacco exposure and the *MUTYH His/His *genotype compared with the *Gln/Gln *genotype showed a significant association with lung cancer risk in smokers, and there was not significantly increased in non-smokers (adjusted OR 3.82, 95%CI 1.22–12.00, p = 0.022 for smokers; adjusted OR 2.60, 95%CI 0.60–11.25, p = 0.200 for non-smokers, respectively). The effect of tobacco exposure and the *OGG1 *Ser326Cys showed also no significant risk for lung cancer.

**Conclusion:**

Our findings suggest that the *MUTYH *Gln324His polymorphism appear to play an important role in modifying the risk for lung cancer in the Japanese population.

## Background

Lung cancer is a well-known cancer that is caused by carcinogens, such as those in tobacco smoke. Tobacco smoke contains many chemical carcinogens and reactive oxygen species, including polycyclic aromatic hydrocarbons. DNA damage induced by these carcinogens or by endogenous metabolic processes can be converted into gene mutations. Recently, in a hospital-based patient-control study, we reported that genetic polymorphisms of *NAT2 *and *CYP1A2 *in metabolic processes contributed to lung cancer susceptibility depending on smoking status in Japanese population [1].

Genetic variation in DNA repair genes are thought to modulate DNA repair capacity and are suggested to be related to cancer risk [2]. The base excision repair (BER) pathway, one of the DNA repair pathways, plays an important role in repairing the DNA damage resulting from chemical alterations of a single base, such as methylated, oxidized, or reduced bases [3]. The most stable product of oxidative DNA damage, 8-oxo-7, 8-dihydro-2'-deoxyguanosine (8-oxoG), causes G:C→T:A transversions, because 8-oxoG pairs with adenine as well as cytosine [4]. In human cells, the proteins that repair these mutations are 8-oxo-guanine glycosylase-1 (OGG1), which is involved in direct repair by 8-oxoG DNA glycosylase, and mutY homolog (MUTYH), which is involved in repair of adenine to 8-oxoG mismatch or that of guanine to 1,2-dihydro-2-oxoadenine (2-OH-A) mismatch due to its glycosylase activity [5,6].

In this study, we focused on *OGG1 *Ser326Cys (rs1052133) and *MUTYH *Gln324His (rs3219489). In some patient-control studies, *OGG1 *Ser326Cys appeared to be associated with an increased risk for lung cancer [7-9], whereas the findings of this association study have been inconsistent [10]. In *MUTYH *gene, it was shown that the inherited variants Tyr165Cys and Gly382Asp have been associated with colorectal tumors in Caucasians, not in East Asians including Japanese [11-13]. The other polymorphism, *MUTYH *Gln324His, have been associated with colorectal tumors in a Japanese population [14,15]. Our recent study found that the *MUTYH *Gln324His constitutes an increased risk of colorectal cancer [16]. To our knowledge, no previous report has examined the effect of *MUTYH *Gln324His with a functional partner of OGG1, for lung cancer and the significant role of base excision repair genes for oxidative damage in relation to smoking. We also investigated two gene variants in lung cancer with the histological subtypes of adenocarcinoma and squamous cell carcinoma; smoking act differently in the development of various histologic types of lung cancer [17]. Therefore, we specifically examined whether two gene polymorphisms, *OGG1 *Ser326Cys and *MUTYH* Gln324His play an interactive role in the risk for lung cancer incidence in relation to the histological subtypes and the smoking status.

## Materials and methods

### Study subjects

The lung cancer patients and controls in this small patient-control study were included in a previous study that investigated the genetic polymorphisms of metabolic enzymes [1]. The 108 lung cancer patients (67 with lung adenocarcinoma, 31 with lung squamous cell carcinoma, and 10 with other carcinomas) were recruited between April 2001 and July 2002 at the Hyogo Medical Center for Adults in Akashi City, Japan. The 121 controls who were selected from outpatients with no current or previous diagnosis of cancer were recruited between November 2002 and March 2003. They suffered mainly from: gastrointestinal disease, hypertension and diabetes. Informed consent was obtained and detailed exposure data on smoking was collected by a personal interview. The study design was approved by the Ethics Review Committee on Genetic and Genomic Research, Kobe University Graduate School of Medicine. Informed consent was obtained from all patients and controls, and all samples were coded after collection of blood and data (questionnaire on smoking habits, etc.). The amount of smoke exposure was calculated as pack-years, the product of the number of years an individual smoked and the average number of cigarettes smoked per day (converted into a standard pack of 20 cigarettes).

### Genotyping

The genomic DNA to be used was isolated for the previous study [1]. The genotype of *OGG1 *Ser326Cys [7] and *MUTYH *Gln324His [16] was determined by PCR-RFLP analysis, as described previously.

### Statistical analysis

Statistical analysis was performed with the SPSS software package (version 14.0 for Windows; SPSS Japan Inc., Tokyo, Japan). Hardy-Weinberg equilibrium was tested using the goodness-of-fit Chi-square test to compare the observed genotype frequencies with the expected genotype frequencies among the control subjects. Associations were expressed as odds-ratios (OR) with 95% confidence interval (95% CI) and p < 0.05 was considered statistically significant. Logistic regression analysis was performed to assess the association between each genotype and lung cancer. ORs, which were computed to estimate the association between certain genotypes and lung cancer, were adjusted for age, gender, and smoking habit (number of pack-years smoked). The subjects were divided into two groups according to pack-years smoked: never-smokers (pack-years = 0) and ever-smokers (pack-years > 0).

## Results

We present the characteristics of lung cancer in Table 1, including 108 patients and 121 controls. There was no difference in the gender distribution (p = 0.491) between males (patients, 65.7%; controls, 61.2%) and females (patients, 34.3%; controls, 38.8%). There was no difference in the average ages (± SD) between patients (65.5 ± 9.4 years) and controls (67.4 ± 6.7 years) (p = 0.078). Non-smokers comprised 29.6% of patients and 45.5% of controls and smokers comprised 68.5% of patients and 49.6% of controls. There was also no difference in the average pack-years (± SD) between patients (33.8 ± 31.7) and controls (25.6 ± 35.1) (p = 0.069). Histological types of the patients were: 67 adenocarcinoma (62.0%), 31 squamous cell carcinoma (28.7%) and 10 others (9.3%).

**Table 1:** Characteristics of lung cancer case and control subjects

		Patients		Controls		
				
Item		n	%	n	%	P-value
Number		108		121		
Gender						
	males	71	65.7	74	61.2	0.491^a^
	females	37	34.3	47	38.8	
Age						
	~64	40	37.0	50	41.3	
	65~69	17	15.7	29	24.0	
	70~74	30	27.8	20	16.5	
	75~	19	17.6	22	18.2	
	unknown	2	1.9	0	0.0	
	Mean ± S.D.	65.5 ± 9.4		67.4 ± 6.7		0.078^b^
Smoking status (Pack-years)						
	Never (Pack-years = 0)	32	29.6	55	45.5	
	Ever (Pack-years > 0)	74	68.5	60	49.6	
	unknown	2	1.9	6	5.0	
	Mean ± S.D.	33.8 ± 31.7		25.6 ± 35.1		0.069^b^
Histological type						
	adenocarcinoma	67	62.0			
	squamous cell carcinoma	31	28.7			
	others	10	9.3			

a: χ^2 ^analysis

b: Student's T-test

Genotyping results of *OGG1 *Ser326Cys and *MUTYH *Gln324His adjusted for gender, age, and smoking habit along with allele frequencies are shown in Table 2. The allele frequencies of the two gene polymorphisms in controls were consistent with the Hardy-Weinberg equilibrium. The crude and adjusted ORs for the *OGG1 Ser/Cys *or *Cys/Cys *genotypes compared with the *Ser/Ser *genotype were not statistically significant. The crude and adjusted ORs for the *MUTYH His/His *genotype compared with *Gln/Gln *genotype showed a increased risk for lung cancer (crude odds ratio [OR] 3.25, 95% confidence interval [95%CI] 1.44–7.36, p = 0.005; adjusted OR 3.03, 95%CI 1.31–7.00, p = 0.010, respectively), whereas there was no significant increase for the *Gln/His *genotype (crude OR 1.39, 95%CI 0.74–2.62, p = 0.309; adjusted OR 1.35, 95%CI 0.70–2.61, p = 0.376, respectively).

**Table 2:** Genotype distribution in lung cancer and Allele frequency

											Allele frequency	
											
Genotype		patients (n = 108)		controls (n = 121)		crude		adjusted			patients	controls
								
		n	%	n	%	OR (95%CI)	P-value	OR (95%CI)^a^	P-value		%	%
*OGG1*												
	Ser/Ser	27	25.0	39	32.2	1.00		1.00		Ser	0.505	0.546
	Ser/Cys	55	50.9	54	44.6	1.47 (0.79–2.73)	0.221	1.52 (0.80–2.91)	0.204	Cys	0.495	0.455
	Cys/Cys	26	24.1	28	23.1	1.34 (0.65–2.77)	0.427	1.47 (0.69–3.12)	0.313			
												
*MUTYH*												
	Gln/Gln	22	20.3	37	30.6	1.00		1.00		Gln	0.468	0.591
	Gln/His	57	52.8	69	57.0	1.39 (0.74–2.62)	0.309	1.35 (0.70–2.61)	0.376	His	0.532	0.409
	His/His	29	26.9	15	12.4	3.25 (1.44–7.36)	0.005	3.03 (1.31–7.00)	0.010			

a: OR adjusted for gender, age, smoking habit

Table 3 summarizes the genotype distribution for lung adenocarcinoma and squamous cell carcinoma, showing the OR adjusted for gender, age, and smoking habits. The crude and adjusted ORs for the *OGG1 Ser/Cys *or *Cys/Cys *genotypes compared with the *Ser/Ser *genotype were not significant for adenocarcinoma and squamous cell carcinoma. The crude ORs for the *MUTYH His/His *genotype compared with *Gln/Gln *genotype showed a significant increase for both adenocarcinoma and squamous cell carcinoma (OR 3.04, 95%CI 1.18–7.82, p = 0.021 for adenocarcinoma; OR 4.11, 95%CI 1.27–13.33, p = 0.019, respectively). The adjusted ORs for the *MUTYH His/His *genotype compared with *Gln/Gln *genotype showed a borderline significant for adenocarcinoma and squamous cell carcinoma (OR 2.50, 95%CI 0.95–6.62, p = 0.065 for adenocarcinoma; OR 3.20, 95%CI 0.89–11.49, p = 0.075 for squamous cell carcinoma, respectively). While, there was no significant increase for the *MUTYH Gln/His *genotype in the histological types.

**Table 3:** Genotype distribution in relation to histological type in lung cancer

Genotype	Adenocarcinoma								Squamous Cell Carcinoma							
		
	patients (n = 67)		controls (n = 121)		crude		adjusted		patients (n = 31)		controls (n = 121)		crude		adjusted	
								
	n	%	n	%	OR (95%CI)^a^	P-value	OR (95%CI)^a^	P-value	n	%	n	%	OR (95%CI)^a^	P-value	OR (95%CI)^a^	P-value
*OGG1*																
Ser/Ser	17	25.4	39	32.2	1.00		1.00		8	25.8	39	32.3	1.00		1.00	
Ser/Cys	33	49.2	54	44.6	1.40 (0.69–2.87)	0.355	1.34 (0.64–2.81)	0.439	16	51.6	54	44.6	1.44 (0.56–3.71)	0.445	1.23 (0.44–3.43)	0.695
Cys/Cys	17	25.4	28	23.1	1.39 (0.61–3.19)	0.434	1.31 (0.56–3.08)	0.530	7	22.6	28	23.1	1.22 (0.40–3.75)	0.730	1.54 (0.45–5.23)	0.491
*MUTYH*																
Gln/Gln	13	19.4	37	30.6	1.00		1.00		6	19.4	37	30.6	1.00		1.00	
Gln/His	38	56.7	69	57.0	1.57 (0.74–3.30)	0.237	1.55 (0.72–3.32)	0.263	15	48.4	69	57.0	1.34 (0.48–3.75)	0.576	1.00 (0.33–3.01)	0.999
His/His	16	23.9	15	12.4	3.04 (1.18–7.82)	0.021	2.50 (0.95–6.62)	0.065	10	32.3	15	12.4	4.11 (1.27–13.33)	0.019	3.20 (0.89–11.49)	0.075

a: OR adjusted for gender, age, smoking habit

The ORs for the combined effect of tobacco exposure (pack-years smoked) and the two polymorphisms, adjusted for gender and age, are shown in Table 4. The crude and adjusted ORs for the *OGG1 Ser/Cys *or *Cys/Cys *genotypes compared with the *Ser/Ser *genotype showed no statistically significant risk in non-smokers and smokers. The crude and adjusted ORs for the *MUTYH *His/His genotype compared with the *Gln/Gln *genotype showed a significant association with lung cancer risk in smokers (crude OR 3.50, 95%CI 1.13–10.83, p = 0.030; adjusted OR 3.82, 95%CI 1.22–12.00, p = 0.022, respectively), and there was not statistically significant in non-smokers (crude OR 3.20, 95%CI 0.81–12.65, p = 0.097; adjusted OR 2.60, 95%CI 0.60–11.25, p = 0.200, respectively). The crude and adjusted ORs for the *MUTYH Gln/His *genotype compared with the *Gln/Gln *genotype showed no statistically significant risk in non-smokers and smokers.

**Table 4:** Genotype distribution in relation to smoking status in lung cancer

	Non-smokers								Smokers							
Genotype	(Pack-years = 0)								(Pack-years > 0)							
		
	patients (n = 32)		controls (n = 55)		crude		adjusted		patients (n = 74)		controls (n = 60)		crude		adjusted	
								
	n	%	n	%	OR (95%CI)^a^	P-value	OR (95%CI)^a^	P-value	n	%	n	%	OR (95%CI)^a^	P-value	OR (95%CI)^a^	P-value
OGG1																
Ser/Ser	5	15.6	14	25.5	1.00		1.00		20	27.0	23	38.3	1.00		1.00	
Ser/Cys	20	62.5	26	47.3	2.15 (0.67–6.98)	0.201	2.49 (0.72–8.57)	0.148	35	47.3	25	41.7	1.61 (0.73–3.54)	0.237	1.53 (0.69–3.40)	0.292
Cys/Cys	7	21.9	15	46.9	1.31 (0.34–5.09)	0.700	1.38 (0.34–5.64)	0.654	19	25.7	12	20.0	1.82 (0.71–4.66)	0.211	1.81 (0.70–4.65)	0.219
*MUTYH*																
Gln/Gln	5	15.6	18	32.7	1.00		1.00		17	23.0	17	28.3	1.00		1.00	
Gln/His	19	59.4	28	50.9	2.44 (0.77–7.71)	0.128	2.06 (0.63–6.76)	0.233	36	48.6	37	61.7	0.97 (0.43–2.20)	0.947	1.07 (0.47–2.46)	0.867
His/His	8	25.0	9	16.4	3.20 (0.81–12.65)	0.097	2.60 (0.60–11.25)	0.200	21	28.4	6	10.0	3.50 (1.13–10.83)	0.030	3.82 (1.22–12.00)	0.022

a: OR adjusted for gender, age

## Discussion

Herein, we report that gene polymorphisms, *OGG1 *Ser326Cys and *MUTYH *Gln324His, of two DNA repair genes in the BER pathway can modulate lung cancer risk in a small case-control study. Our results indicated that lung cancer risk was found to be 3-fold in individuals with the homozygous *His/His *genotype of *MUTYH *Gln324His (95%CI 1.31–7.00, p = 0.010), whereas that was not with that of *OGG1 *Ser326Cys. The *MUTYH His/His *genotype also show a borderline significant risk for both adenocarcinoma and squamous cell carcinoma. Moreover, a joint effect between tobacco smoking and the *MUTYH His/His *genotype for the risk of lung cancer was statistically increased in smokers, whereas that was not in non-smokers.

We found that no significant effect was apparent between *OGG1 *Ser326Cys and lung cancer risk, in combination to smoking status. It has been reported that the *OGG1 Cys *allele in Japanese patients is associated with an increased risk for lung cancer [8,9]. The variant OGG1 is deficient in its catalytic activity, was not stimulated by the AP endonuclease [18]. A recent report has suggested that *OGG1 *Ser326Cys is not associated with lung cancer by meta-analysis [10]. Therefore, our finding in a Japanese population is consistent with the results from the meta-analysis study.

On the other hand, we found that the *MUTYH His/His *genotype was significantly associated with increased risk of lung cancer. Previous study has shown that the identified variants of the *MUTYH *gene, containing Gln324His, were unlikely to predispose significantly to the risk for lung cancer in Caucasians [19]. The discrepancy between this study and ours might reflect the differences in genetic background, carcinogen exposure in different populations or sample sizes. Recent study has reported that the MUTYH enzyme activity in Gln324His polymorphism was only 66% active from the substrates compared with the wild type [20]. It was reported that the 2-OH-A level compared to repair of adenine opposite 8-oxo-G was increased in human cancerous tissues compared to normal tissues [21]. Therefore, it is also possible that the MUTYH enzyme having *324His *variation may have partially a reduced activity in repair of 2-OH-A opposite guanine. This suggested that *MUTYH *Gln324His might also be associated with risk for lung cancer, related to the decreased MUTYH enzyme activity.

In different histological types of lung cancer, *MUTYH His/His *genotype was a significantly borderline association for both adenocarcinoma and squamous cell carcinoma, that suggested a potential interaction between this polymorphism and lung cancer risk regardless these subtypes. Moreover, the result of the joint effect between tobacco smoking and *MUTYH His/His *genotype for the risk of lung cancer was a significant increase in smokers, whereas that was not in non-smokers. If the sample size had been larger, the result in non-smokers might have been significant. This finding suggested that the effect of *MUTYH *Gln324His for lung cancer risk is not different between smoking habits.

In conclusion, these results suggest that the *MUTYH *Gln324His polymorphism appear to play an important role in modifying the risk for lung cancer in the Japanese population. To the best of our knowledge, our study is the first case-control study to evaluate the association between the *MUTYH *Gln324His and lung cancer risk in Japanese. The *MUTYH*Gln324His polymorphism may be one of useful markers of genetic susceptibility to lung cancer and require further verification as predictive biomarkers in a larger study population.

## Competing interests

The authors declare that they have no competing interests.

## Authors' contributions

AM, KO and JT plan the study made all coordination and was involved in the laboratory processing. YO, NI, KY and MK participated in the study and performed the statistical analysis. AT, YT, KS and NT carried out handling the samples. All authors read and approved the final version of manuscript.
